# A Longitudinal Study of Peripubertal Serum Organochlorine Concentrations and Semen Parameters in Young Men: The Russian Children’s Study

**DOI:** 10.1289/EHP25

**Published:** 2016-10-07

**Authors:** Lidia Mínguez-Alarcón, Oleg Sergeyev, Jane S. Burns, Paige L. Williams, Mary M. Lee, Susan A. Korrick, Luidmila Smigulina, Boris Revich, Russ Hauser

**Affiliations:** 1 Department of Environmental Health, Harvard T.H. Chan School of Public Health, Boston, Massachusetts, USA; 2 Department of Genomics and Human Genetics Vavilov Institute of General Genetics, Russian Academy of Sciences, Moscow, Russia; 3 Chapaevsk Medical Association, Chapaevsk, Samara Region, Russia; 4 Department of Biostatistics, and; 5 Department of Epidemiology, Harvard T.H. Chan School of Public Health, Boston, Massachusetts, USA; 6 Pediatric Endocrine Division, Departments of Pediatrics and Cell & Developmental Biology, University of Massachusetts Medical School, Worcester, Massachusetts, USA; 7 Channing Division of Network Medicine, Department of Medicine, Brigham and Women’s Hospital, Harvard Medical School, Boston, Massachusetts, USA; 8 Institute for Forecasting, Russian Academy of Sciences, Moscow, Russia

## Abstract

**Background:**

Exposures to endocrine-disrupting chemicals during critical phases of testicular development may be related to poorer semen parameters. However, few studies have assessed the association between childhood organochlorine (OC) exposure and adult semen parameters.

**Objective:**

We examined whether peripubertal serum OC concentrations are associated with semen parameters among young Russian men.

**Methods:**

From 2003 through 2005, 516 boys were enrolled at age 8–9 years and followed for up to 10 years. Serum OCs were measured in the enrollment samples using high-resolution mass spectrometry. At 18–19 years, 133 young men provided 1 or 2 semen samples (256 samples) collected approximately 1 week apart, which were analyzed for volume, sperm concentration, and motility. Unadjusted and adjusted linear mixed models were used to examine the associations of quartiles of lipid-standardized concentrations of dioxins [2,3,7,8-tetrachlorodibenzo-*p*-dioxin (TCDD), polychlorinated dibenzo-*p*-dioxins (PCDDs)], furans, polychlorinated biphenyls (PCBs), and corresponding toxic equivalents (TEQs) with semen parameters.

**Results:**

The median (range) for TCDD was 2.9 (0.4–12.1) pg/g lipid and PCDD TEQ was 8.7 (1.0–36.0) pg TEQ/g lipid. Higher quartiles of TCDD and PCDD TEQs were associated with lower sperm concentration, total sperm count, and total motile sperm count (*p*-trends ≤ 0.05). The highest quartile of peripubertal serum TCDD concentrations was associated with a decrease (95% CI) of 40% (18, 66%), 29% (3, 64%), and 30% (2, 70%) in sperm concentration, total sperm count, and total motile sperm count, respectively, compared with the lowest quartile. Similar associations were observed for serum PCDD TEQs with semen parameters. Serum PCBs, furans, and total TEQs were not associated with semen parameters.

**Conclusion:**

Higher peripubertal serum TCDD concentrations and PCDD TEQs were associated with poorer semen parameters.

**Citation:**

Mínguez-Alarcón L, Sergeyev O, Burns JS, Williams PL, Lee MM, Korrick SA, Smigulina L, Revich B, Hauser R. 2017. A longitudinal study of peripubertal serum organochlorine concentrations and semen parameters in young men: the Russian Children’s Study. Environ Health Perspect 125:460–466; http://dx.doi.org/10.1289/EHP25

## Introduction

Over the past several decades, numerous studies have explored whether semen parameters have declined (Carlsen et al. 1992; Swan et al. 2000), and whether there are geographical differences in semen parameters both between (Jørgensen et al. 2001, 2002) and within countries (Swan et al. 2003). Recent literature has shown that serum concentrations of organochlorines (OCs), including dioxins, furans, and polychlorinated biphenyls (PCBs), are associated with decreased semen parameters (Faure et al. 2014; Meeker and Hauser 2010; Mocarelli et al. 2008, 2011; Paoli et al. 2015; Toft et al. 2006). Despite efforts to limit dioxin emissions and longstanding bans on PCB manufacture and use, there is still ongoing exposure through diet because these compounds bioconcentrate in the food chain due to their lipophilic properties and long half-lives (Schecter et al. 2001).

Among epidemiologic studies on OCs and semen parameters, the only one that explored childhood exposure and adult semen parameters was in Seveso, Italy, where an explosion in 1976 at a trichlorophenol manufacturing plant released up to 30 kg of 2,3,7,8-tetrachlorodibenzo-*p*-dioxin (TCDD) (Mocarelli et al. 2008). The authors investigated the relationship of serum TCDD concentrations measured from blood samples taken in 1976 during childhood (1–9 years), puberty (10–17 years), or young adult life (18–26 years) with semen parameters and male reproductive hormones measured 22 years later. They did not measure other dioxins, furans or PCBs. Mocarelli and colleagues found that acute high exposure to TCDD in childhood (1–9 years), but not during puberty (10–17 years) or adulthood (18–26 years), was associated with poorer semen parameters later in adulthood. These compelling results were key data in the U.S. Environmental Protection Agency (EPA) risk assessment for dioxins (U.S. EPA 2009). These results suggested that during childhood, when the testes are still immature, the activation of aryl hydrocarbon receptors (AhR) in the testes by TCDD may interfere with maturation of the seminiferous tubules and spermatogenesis and demonstrates that the juvenile reproductive system may be particularly vulnerable to TCDD exposure (Woodruff et al. 2010).

Given the importance of childhood exposures on reproductive health later in life, we conducted a prospective cohort study of Russian boys with a wide range of exposure to dioxins, furans, and PCBs stemming from environmental contamination of their community. Specifically we assessed the associations of peripubertal (measured at age 8–9 years) serum concentrations of dioxins, furans, and PCBs with semen parameters in young healthy men measured approximately 10 years later.

## Methods

### Study Population

**Figure 1:**
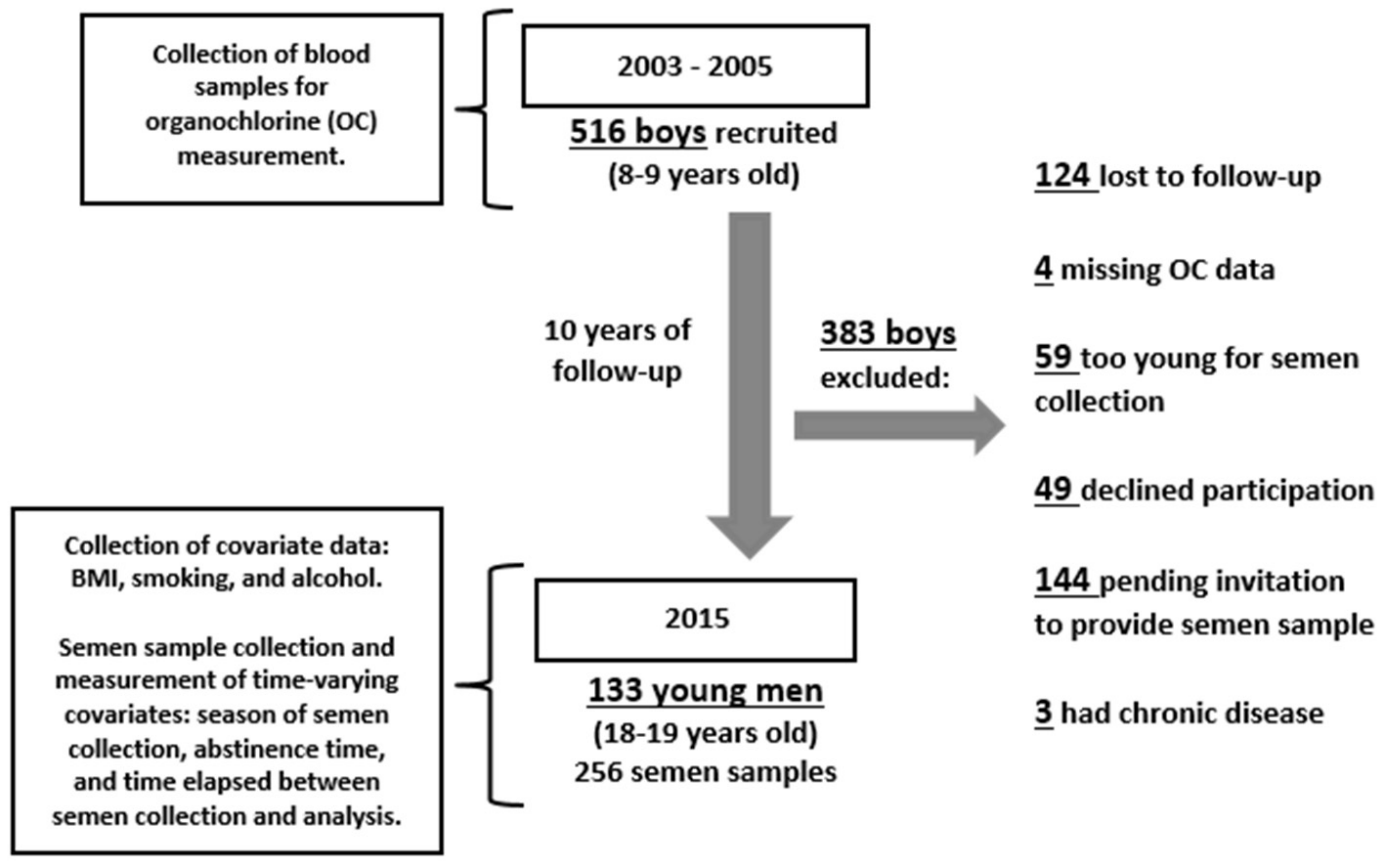
Flow diagram of the Russian Children’s Study. Note: Information on BMI, smoking, and alcohol consumption was collected at the same visit year as the semen collection for 84 (63%) men, and within 3 years before semen collection for 49 (37%) men.

The Russian Children’s Study is an ongoing prospective study of 516 males (Hauser et al. 2008; Williams et al. 2010). Once enrolled at age 8–9 years, each boy underwent a physical examination, provided a blood sample for OC measurement, and together with his mother or guardian, completed health, lifestyle, and dietary questionnaires. Annual follow up examinations were conducted and questionnaires were completed. Of the original cohort of 516 boys, 124 (24%) were lost to follow-up by their 10th annual follow-up visit at age 18–19 years, 59 (11%) were too young for semen collection, 49 (15%) declined to participate in the semen study, 144 (28%) were pending (did not respond yet to invitation, temporarily relocated, or not yet sexually mature based on Tanner Stages and testicular volume), 4 had missing OC data, and 3 were excluded due to chronic disease. At ages 18–19 years, 133 young men who had serum OC concentrations measured at age 8–9 years and provided 1 or 2 semen samples collected approximately 1 week apart (256 samples) were included in this analysis (Figure 1).

The study was approved by the Human Studies Institutional Review Boards of the Chapaevsk Medical Association (Chapaevsk, Russia); Harvard T.H. Chan School of Public Health, Brigham and Women’s Hospital (Boston, MA, USA), and University of Massachusetts Medical School (Worcester, MA, USA). At enrollment, the parent or guardian signed an informed consent, and each boy signed an assent before participation. At ≥ 18 years of age, the young man signed a consent form before providing the two semen samples.

### Semen Parameters Assessment

The subjects’ self-reported information about abstinence period, fever, and any illnesses within the previous month was collected before semen sampling. Semen samples were provided by masturbation in a study room near the Andrology Laboratory and kept at 37°C in an incubator until semen evaluation, which began within 1 hr after ejaculation (analysis for 88% of the samples began within 30 min). One hundred twenty-three men (92%) provided two semen samples collected approximately 1 week apart, and 10 men (8%) provided one semen sample. The actual abstinence period was calculated from the date and time of previous ejaculation and the date and time of delivery of semen sample recorded by a technician.

Semen analysis was performed at the Andrology Laboratory according to the criteria recently updated (Björndahl et al. 2010) by the Nordic Association for Andrology (NAFA) and European Society of Human Reproduction and Embryology–Special Interest Group in Andrology (ESHRE-SIGA) (Kvist and Björndahl 2002). All samples were assessed by one technician (L.S.) who was blinded to the serum OC concentration. Semen volume was measured using a 1-, 5-, or 10-mL disposable pipette. For sperm motility assessment, 10 μL of well-mixed semen was placed on a clean glass slide kept at 37°C and covered with a 22 × 22 mm coverslip. The slide was placed on the heated stage of a microscope at 37°C and immediately examined at 400× magnification in duplicate. At least 200 sperm per slide were classified as the four World Health Organization (WHO) classes: rapidly progressive motile (class A), slowly progressive motile (class B), locally motile (class C) or immotile (class D), taking the average value for duplicate measures (WHO 1999). Percent motile sperm was defined as the sum of WHO classes A, B, and C. Sperm concentration was measured using an Improved Neubauer Chamber Hemacytometer viewed at phase contrast (200× magnification).

### Organochlorine Exposure Assessment

Fasting blood samples were collected at the initial visit (when boys were 8–9 years old), and the serum fraction was stored at –35°C until shipment for analysis at the National Center for Environmental Health at the Centers for Disease Control and Prevention (CDC; Atlanta, GA, USA). Analytes included 7 polychlorinated dibenzo-*p*-dioxins (PCDDs, or dioxins), 10 polychlorinated dibenzofurans (PCDFs, or furans), 4 co-planar PCBs (co-PCBs), 6 mono-*ortho*–substituted PCBs, and 31 other PCBs (non-dioxin-like PCBs) (Burns et al. 2009).

For dioxin-like analytes, sera, method blanks, and quality control samples (aliquots of pooled bovine sera) were spiked with a mixture of ^13^C_12_-labeled PCDDs/PCDFs and co-PCBs as internal standards, and serum analytes were isolated by solid phase extraction (SPE) followed by a multicolumn automated cleanup and enrichment procedure (Turner et al. 1997). Analytes were separated on a DB-5 MS capillary column (Phenomenex, Torrance, CA, USA) and quantified using selected-ion-monitoring (SIM) high-resolution (10,000 resolving power) mass spectrometry (HRGC-ID/HRMS; Thermo Electron North America, LLC, West Palm Beach, FL, USA) (Patterson et al. 1987). Quantification was by isotope dilution MS using calibration standards containing ^13^C_12_-labeled and unlabeled analytes. A similar approach was used for mono-*ortho* and non-dioxin-like PCBs (Barr et al. 2003). Samples were spiked with ^13^C_12_-labeled PCBs, extracted by either large (Turner et al. 1997) or small (Sjödin et al. 2004) volume SPE, and analyzed using HR GC/MS in SIM (Barr et al. 2003).

For all analyses, quality control sample coefficients of variation combining between-run and within-run reproducibility were generally < 15%. All concentrations were expressed on a per-lipid basis, with serum total cholesterol and triglycerides measured enzymatically, and total lipids were calculated using the Phillips equation (Phillips et al. 1989). Congener concentrations below the limit of detection (LOD) were assigned the sample-specific LOD divided by the square root of 2 (Baccarelli et al. 2005).

### Statistical Analysis

Dioxin toxic equivalents (TEQs) were calculated on a lipid basis using the 2005 WHO toxic equivalency factors to weigh the potency of each congener relative to TCDD before summation (Van den Berg et al. 2006). Although our *a priori* hypothesis focused on TCDD, we also explored the association of eight additional exposure metrics with semen parameters. These included *1*) total (summed) TEQ measures (pg TEQ/g lipid) for combined dioxin, furan, co-planar PCB, and mono-*ortho* PCB congeners; *2*–*4*) total (summed) TEQs (pg TEQ/g lipid) for each of the dioxins, furans, and co-PCBs; *5*–*7*) total (summed) concentrations (pg/g lipid) for each of the dioxins (ΣPCDD), furans (ΣPCDF), and co-PCBs (ΣCo-PCB); and *8*) total (summed) concentrations of non-dioxin-like PCBs, including mono-*ortho*–substituted PCBs (ΣPCBs) (ng/g lipid). OC measures were categorized into quartiles because of potential nonlinear associations.

We first summarized participant characteristics using medians and interquartile ranges (IQR) for continuous variables, and number and percentages for categorical variables. Linear mixed models were used to examine the relation between OC exposure and semen parameters with adjustment for potential confounders; within-person correlations in semen parameters across repeated samples were accounted for using random intercepts. We compared semen parameters (total sperm count, sperm concentration, percent motile sperm, total motile sperm count, and semen volume) for men with higher quartiles of serum OC concentrations to those within the lowest quartile. Total sperm count (volume × sperm concentration) and total motile sperm count (total sperm count × percent motile sperm) were calculated. Total sperm count, sperm concentration, and total motile sperm count were log-transformed to approximate a normal distribution. Results for these parameters were back-transformed to allow presentation of results in the original scale. Population marginal means (Searle et al. 1980) were utilized to present marginal population average semen parameters adjusted for the covariates (at the mean level for continuous variables and for categorical variables at a value weighted according to their frequencies) in the model. Tests for linear trends were conducted using quartile of serum OC concentrations as ordinal levels.

Potential confounding factors that were included in the models were selected primarily based on *a priori* evidence from the literature but supported empirically by associations with one or more of the semen parameters and/or serum OCs. In addition, we decided to include abstinence time regardless of statistical significance since this is a well-known predictor of most semen quality parameters, and thus can improve the precision of the exposure estimates in the model (Schisterman et al. 2009). Based on these criteria, all models were adjusted for body mass index (BMI) from the most recent physical examination, smoking status (yes vs. no, based on the response to the question “Have you smoked a cigarette, even a few puffs, within the past year?”), alcohol consumption (yes vs. no, based on the response to the question “Have you drunk alcohol in the last year, including beer?”), season of semen collection (autumn or winter vs. spring or summer), and abstinence time (< 2 days, 2–5 days, ≥ 5 days). Percent of motile sperm and total motile sperm count models were further adjusted for the time elapsed between semen collection and semen parameter analysis. Information on BMI, smoking status, and alcohol consumption was collected at the same visit year as the semen collection for 84 (63%) men, and within 3 years before semen collection for the remaining 49 (37%) men. BMI, smoking status, and alcohol consumption were unchanged between the two semen samples collected approximately 1 week apart; season, abstinence time, and time elapsed between semen collection and analysis were considered as time-varying measures for each semen sample. We analyzed the data using SAS (version 9.2; SAS Institute Inc., Cary, NC, USA), and two-sided *p*-values ≤ 0.05 were considered statistically significant.

## Results

At the time of semen collection, study participants were young men with median age (IQR) = 18.3 (18.1–18.7) years, 100% Caucasian, and the median (IQR) BMI was 21.0 (19.2–23.2) kg/m^2^ (Table 1). Fifty-one percent of the participants had smoked cigarettes (self-reported), and 68% had consumed alcohol (parental report) within the past year. One hundred thirty-three semen samples (52%) were above NAFA-ESHRE reference values for sperm counts (≥ 80 million) and motility (≥ 60%) (Björndahl et al. 2010). The median (IQR) values for sperm parameters were 51.3 million/mL (26.6–78.8) for sperm concentration; 127 million (61.0–222) for total sperm count; and 64.0% (57.0–68.0) for sperm motility. Median (IQR) abstinence time was just under 3 days (2–6) (Table 1).

**Table 1:** Demographic characteristics and semen parameters of 133 young men contributing 256 semen samples in the Russian Children’s Study.

Characteristic	Median (IQR) or *n* (%)
Demographic characteristics^*a*^
Age (years )	18.3 (18.1–18.7)
Body mass index (kg/m^2^)	21.0 (19.2–23.2)
Smoking status^*b*^	68 (51)
Alcohol consumption^*c*^	90 (68)
Semen parameters^*d*^
Volume (mL)	2.4 (1.8–3.5)
Sperm concentration (million/mL)	51.3 (26.6–78.8)
Total sperm count (million)	127 (61.0–222.0)
Sperm motility (A + B + C)^*e*^ (%)	64.0 (57.0–68.0)
Total motile sperm count (million)	80.5 (35.8–141.0)
Abstinence time (days)	2.9 (2.0–6.0)
IQR, interquartile range. ^***a***^Assessed at the time of semen collection (or at visit closest in time). ^***b***^Question was “In the past year, have you smoked a cigarette, even a few puffs?” In some cases, the questionnaire was filled out up to 3 years before the semen sample was collected. ^***c***^Question was “Have you drunk alcohol in the last year, including beer?” In some cases, the questionnaire was filled out up to 3 years before the semen sample was collected. ^***d***^Two semen samples were collected from 123 (93%) young men. ^***e***^This measure includes rapidly progressive motile (class A), slowly progressive motile (class B), and locally motile (class C).	

Serum concentrations of dioxins, furans, and PCBs among participants at ages 8–9 years are presented in Table 2. The median (range) values for TCDD and PCDD TEQs were 2.9 (0.4–12.1) pg/g lipid and 8.7 (1.0–36.0) pg TEQ/g lipid, respectively. Sixteen samples (12%) were below the LOD for TCDD. The median (range) of total serum TEQs was almost three times higher than levels among European children of similar age (Table 2) (Leijs et al. 2008; Link et al. 2005). The correlation between TCDD and PCDD TEQs was *r* = 0.78 (*p* < 0.01) and between PCDD TEQs and total TEQs was *r* = 0.89 (*p* < 0.01). The correlation between total TEQs and co-PCB TEQs was *r* = 0.78 (*p* < 0.01). Correlations among the dioxin and PCB congeners were lower (*r* = 0.42–0.57, *p* < 0.01) (data not shown). When we compared baseline serum organochlorine concentrations adjusted by birth year between those young men who contributed semen samples and those who did not, there were no significant differences (data not shown).

**Table 2:** Serum concentrations and TEQs for dioxins, furans, and PCBs measured at study enrollment (age 8–9 years of age) for 133 young men in the Russian Children’s Study.

Toxic equivalent/concentration	Min	Percentile			Max
		25th	50th	75th	
TEQs (pg TEQ/g lipid)
TCDD^*a*^	0.35	1.77	2.9	4.2	12.1
PCDD TEQ	0.95	5.69	8.7	13.3	36.0
PCDF TEQ	0.55	3.20	4.8	7.1	50.6
Co-PCB TEQ^*b*^	0.52	4.66	6.9	10.0	67.2
Total TEQ^*c*^	1.88	16.8	21.9	33.3	107
Concentration (pg/g lipid)
PCDD	37.6	115	157	199	1,237
PCDF	14.4	29.4	44.5	63.3	406
Co-PCB^*d*^	62.5	131	188	273	965
Concentration (ng/g lipid)
ΣPCBs^*e*^	58.3	152	235	352	1,500
^***a***^Average limit of detection (LOD) for TCDD was 0.60 (pg TEQ/g lipid); 16 samples (12%) were below LOD for TCDD. ^***b***^Sum of co-planar PCB TEQs [International Union of Pure and Applied Chemistry (IUPAC) congeners: 77, 81, 126, 169]. ^***c***^Sum of TEQ measures for combined dioxin, furan, co-PCB and mono-*ortho* PCB congeners. ^***d***^Sum of co-planar PCB concentrations (IUPAC congeners: 77, 81, 126, 169). ^***e***^Sum of non-co-planar PCBs (IUPAC congeners: 18, 28, 52, 49, 44, 74, 66, 101, 99, 87, 110, 118, 105, 151, 149, 146, 153, 138/158, 128, 167, 156, 157, 178, 187, 183, 177, 172, 180, 170, 189, 201, 196/203, 195, 194, 206).					

**Figure 2:**
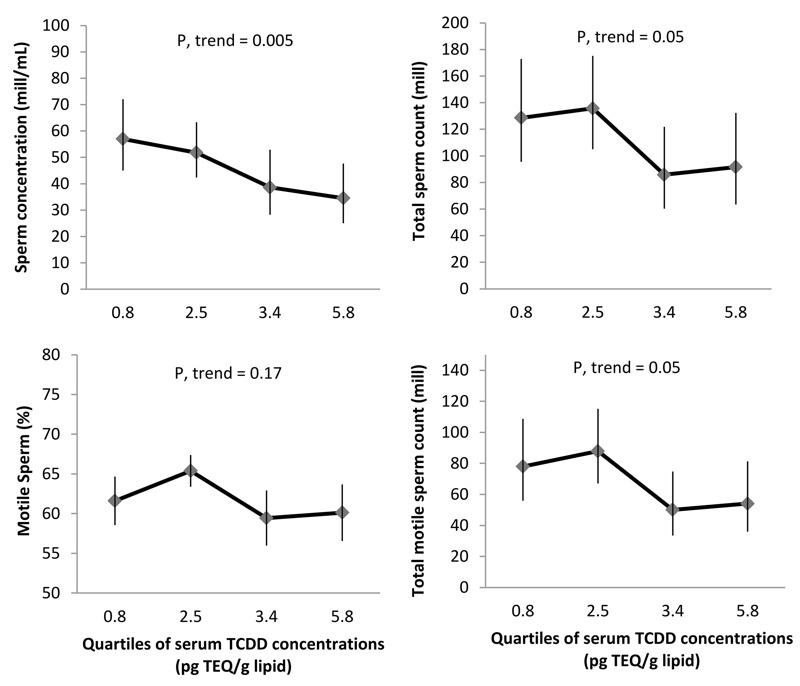
Adjusted mean semen parameters among 133 men (contributing 256 semen samples) from the Russian Children’s Study, by childhood serum TCDD concentrations. Data are presented as predicted marginal means (95% confidence intervals) by quartiles of TCDD concentrations (represented by the medians) adjusted for BMI, smoking status, alcohol drinker, season of sample collection, and abstinence time at the mean level of continuous covariates and adjusted for frequency of categorical measures. Motile sperm and total motile sperm count models were further adjusted by time elapsed between semen collection and analysis.

**Figure 3:**
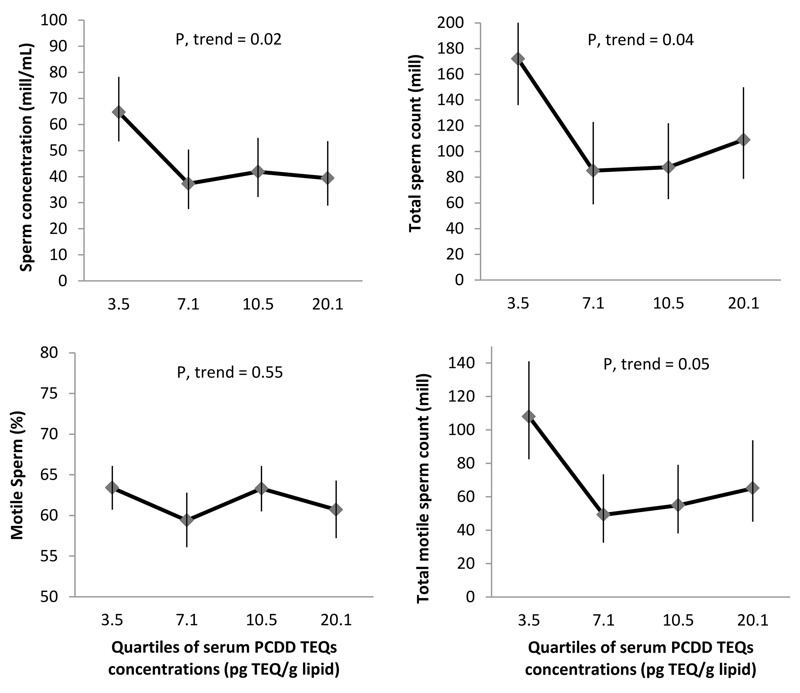
Adjusted mean semen parameters among 133 men (contributing 256 semen samples) in the Russian Children’s Study, by childhood serum PCDD TEQs. Data are presented as predicted marginal means (95% confidence intervals) by quartiles of PCDD TEQs levels (represented by the medians) adjusted for BMI, smoking status, alcohol drinker, season of sample collection, and abstinence time at the mean level of continuous covariates and adjusted for frequency of categorical measures. Motile sperm and total motile sperm count models were further adjusted by time elapsed between semen collection and analysis.

Higher serum TCDD and PCDD TEQs were associated with significantly lower semen parameters 10 years later in both unadjusted models (see Table S1) and models adjusted for BMI, smoking status, alcohol intake, season, and abstinence time (Figures 2 and 3 and Table 3). In adjusted models, on average, men in the highest quartile of serum TCDD TEQs had 40% lower sperm concentration (*p*-trend = 0.005), 29% lower total sperm count (*p*-trend = 0.05), and 30% lower total motile sperm count (*p*-trend = 0.05), compared to those in the lowest quartile (Figure 2). Similarly, men in the highest quartile of serum PCDD TEQs had a decrease of 39% in sperm concentration (*p*-trend = 0.02), 36% in total sperm count (*p*-trend = 0.04), and 40% in total motile sperm count (*p*-trend = 0.05), compared with the lowest quartile of PCDD TEQs (Figure 3).

**Table 3:** Multivariable adjusted mean semen parameters by quartiles (Q)*^a^* of serum dioxins, furans, and PCBs among 133 young men in the Russian Children’s Study contributing 256 semen samples.

Toxic equivalent/concentration	Volume (mL)	Sperm concentration (million/mL)	Total sperm count (million)	Motile sperm (%)	Total motile sperm count (million)
TEQs (pg TEQ/g lipid)
TCDD
Q1 (0.35–1.70)	2.7 (2.2, 3.2)	57.0 (45.0, 72.1)	128 (95.6, 173)	61.6 (58.6, 64.7)	78.0 (56.0, 109)
Q2 (1.77–2.45)	2.9 (2.5, 3.4)	51.8 (42.4, 63.3)	136 (105.0, 175)	65.4 (63.4, 67.4)	87.9 (67.1, 115)
Q3 (3.00–3.40)	2.6 (2.1, 2.9)	38.6 (28.2, 52.9)*	85.8 (60.4, 122)	59.5 (56.0, 62.9)	50.1 (33.5, 74.8)
Q4 (4.40–5.80)	3.1 (2.5, 3.7)	34.5 (25.0, 47.7)*	91.6 (63.5, 132)	60.1 (56.6, 63.7)	54.1 (36.0, 81.4)
*p*-trend	0.55	0.005	0.05	0.17	0.05
PCDD TEQ
Q1 (0.95–5.62)	3.2 (2.7, 3.6)	64.7 (53.5, 78.2)	172 (136.0, 217)	63.4 (60.7, 66.1)	108.0 (82.5, 141)
Q2 (5.69–8.42)	2.6 (2.1, 3.1)	37.3 (27.6, 50.4)*	85.0 (58.9, 123)*	59.4 (56.1, 62.8)	49.2 (32.6, 73.5)*
Q3 (8.68–13.3)	2.4 (2.1, 2.8)*	41.9 (32.2, 54.8)*	87.7 (63.0, 122)*	63.3 (60.5, 66.1)	54.9 (38.1, 79.1)*
Q4 (13.7–36.0)	3.2 (2.6, 3.8)	39.4 (28.9, 53.6)*	109 (78.7, 150)*	60.7 (57.2, 64.3)	65.1 (45.1, 93.8)*
*p*-trend	0.89	0.02	0.04	0.55	0.05
PCDF TEQ
Q1 (0.55–3.20)	2.9 (2.6, 3.4)	49.3 (36.4, 66.7)	128 (93.2, 176)	63.4 (60.8, 65.9)	80.3 (56.6, 114)
Q2 (3.29–4.66)	2.3 (1.9, 2.8)	43.3 (32.3, 58.0)	83.1 (57.2, 121)	59.3 (55.7, 62.9)	48.1 (31.8, 72.6)
Q3 (4.76–6.87)	3.1 (2.5, 3.6)	39.1 (30.9, 49.6)	103 (76.7, 140)	61.1 (58.2, 63.9)	62.3 (44.6, 87.2)
Q4 (7.10–50.6)	3.0 (2.5, 3.6)	47.8 (36.2, 63.1)	126 (94.5, 168)	63.0 (59.6, 66.5)	78.2 (56.5, 108)
*p*-trend	0.48	0.78	0.82	0.90	0.82
Co-PCB TEQ
Q1 (0.52–4.63)	2.8 (2.3, 3.4)	56.5 (44.0, 72.6)	131 (97.6, 175)	63.1 (60.3, 66.0)	81.9 (59.6, 112)
Q2 (4.66–6.87)	2.9 (2.5, 3.3)	36.9 (26.2, 51.8)	95.6 (64.1, 142)	60.8 (57.8, 63.7)	57.0 (36.5, 89.0)
Q3 (6.88–9.97)	2.8 (2.2, 3.3)	37.4 (27.9, 50.2)	88.4 (62.2, 125)	62.1 (58.6, 65.6)	53.7 (36.0, 80.1)
Q4 (10.1–67.2)	2.9 (2.4, 3.4)	51.4 (40.1, 65.9)	127 (95.3, 168)	60.9 (57.3, 64.6)	76.0 (54.7, 106)
*p*-trend	0.89	0.73	0.88	0.47	0.77
Total TEQ
Q1 (4.88–16.8)	3.0 (2.5, 3.5)	51.9 (38.3, 70.4)	131 (94.4, 181)	61.8 (58.7, 64.9)	80.4 (55.5, 116)
Q2 (17.0–21.4)	2.6 (2.2, 3.1)	38.9 (28.7, 52.6)	85.9 (57.9, 128)	61.4 (58.4, 64.3)	51.8 (33.8, 79.4)
Q3 (21.7–32.5)	2.9 (2.4, 3.5)	42.1 (33.9, 52.2)	102 (78.2, 132)	61.2 (58.1, 64.4)	60.8 (45.2, 82.0)
Q4 (33.3–107)	2.8 (2.3, 3.3)	44.8 (33.4, 60.2)	112 (82.4, 151)	61.9 (58.1, 65.6)	67.7 (47.8, 95.9)
*p*-trend	0.84	0.61	0.68	0.99	0.68
Concentration (pg/g lipid)
PCDD
Q1 (37.6–115)	2.9 (2.4, 3.3)	52.0 (39.4, 68.7)	130 (94.5, 180)	64.3 (61.9, 66.8)	83.1 (58.8, 118)
Q2 (118–157)	2.6 (2.0, 3.2)	43.2 (33.9, 55.0)	91.0 (65.8, 126)	58.9 (55.7, 62.0)	52.6 (37.0, 75.7)
Q3 (158–200)	3.3 (2.7, 3.8)	37.6 (28.0, 50.6)	108 (76.8, 151)	63.2 (60.4, 66.0)	67.2 (46.0, 98.3)
Q4 (201–1,237)	2.7 (2.2, 3.2)	47.2 (35.9, 62.1)	109 (81.3, 146)	60.4 (56.7, 64.0)	64.3 (46.3, 89.3)
*p*-trend	0.81	0.48	0.59	0.30	0.49
PCDF
Q1 (14.4–29.2)	2.7 (2.2, 3.1)	53.1 (38.9, 72.5)	122 (86.2, 173)	63.7 (61.1, 66.3)	76.8 (53.1, 111)
Q2 (29.4–43.6)	2.6 (2.2, 3.0)	41.8 (32.1, 54.2)	89.6 (63.2, 127)	60.3 (57.0, 63.6)	53.0 (35.6, 78.9)
Q3 (44.5–63.0)	3.5 (2.9, 4.1)	37.2 (28.2, 48.9)	112 (80.8, 155)	60.0 (56.5, 63.3)	65.9 (45.4, 95.0)
Q4 (63.3–405)	2.6 (2.2, 3.0)	48.6 (37.2, 63.4)	115 (84.9, 155)	63.0 (59.8, 66.2)	71.3 (51.0, 100)
*p*-trend	0.47	0.60	0.93	0.79	0.98
Co-PCB
Q1 (62.5–126)	2.6 (2.1, 3.1)	59.7 (45.8, 77.8)	128 (92.5, 179)	62.5 (59.4, 65.6)	79.1 (54.9, 114)
Q2 (130–184)	2.6 (2.2, 3.0)	39.3 (28.8, 53.7)	88.4 (61.8, 126)	61.5 (58.6, 64.4)	53.6 (36.0, 79.7)
Q3 (187–274)	3.3 (2.7, 3.8)	38.9 (29.7, 51.0)	108 (78.0, 149)	61.4 (58.6, 64.2)	65.0 (45.6, 92.7)
Q4 (275–965)	3.0 (2.5, 3.5)	43.9 (34.4, 56.1)	114 (86.3, 151)	61.5 (57.8, 65.1)	69.1 (49.9, 95.7)
*p*-trend	0.09	0.11	0.79	0.67	0.75
Concentration (ng/g lipid)
ΣPCBs
Q1 (58.3–151)	2.9 (2.4, 3.5)	52.5 (39.8, 69.3)	122 (87.4, 171)	62.6 (59.6, 65.7)	79.9 (55.9, 114)
Q2 (152–236)	2.6 (2.1, 3.0)	47.4 (34.8, 64.5)	103 (69.8, 152)	62.5 (59.9, 64.9)	65.1 (42.9, 98.8)
Q3 (239–352)	2.7 (2.3, 3.2)	33.8 (25.8, 44.3)*	84.3 (61.8, 115)*	61.5 (57.9, 65.1)	48.5 (33.4, 70.4)
Q4 (356–1,500)	3.0 (2.4, 3.6)	45.3 (34.3, 59.9)	110 (79.4, 152)	59.6 (55.9, 63.3)	68.6 (49.7, 94.7)
*p*-trend	0.81	0.24	0.47	0.19	0.36
^***a***^Data are presented as predicted estimates (95% confidence intervals) adjusted for BMI, smoking status, alcohol drinker, season, and abstinence time at the mean level of continuous covariates and adjusted for frequency of categorical measures. Motile sperm and total motile sperm count models were further adjusted by time to start semen analysis. *p < 0.05.					

There were no significant associations for summed concentrations of PCDDs, PCDFs, co-PCBs, or ΣPCBs with semen parameters in unadjusted (see Table S1) or adjusted models (Table 3). PCDF TEQs, co-PCB TEQs, or total TEQs were also not significantly associated with semen parameters in unadjusted (see Table S1) or adjusted models (Table 3).

## Discussion

Our prospective cohort study showed that higher peripubertal serum TCDD and PCDD TEQs were associated with lower sperm concentration, total sperm count, and total motile sperm count measured 10 years later in healthy young men. Serum TCDD and PCDD TEQs were not associated with percent motile sperm, so the association with total motile count was largely driven by the association with total sperm count. We did not observe associations of semen parameters with serum concentrations of PCDDs, PCDFs, co-PCBs, or ΣPCBs, nor with PCDF TEQs, co-PCB TEQs, or total TEQs. The lack of association of semen parameters with total TEQs was surprising given the high correlation between PCDD TEQs and total TEQs. However, this might be explained by the fact that PCDDs account for slightly less than 40% of the total TEQs (Burns et al. 2009). This suggests that the associations we found may be more specific to PCDD TEQs than to overall TEQs, which also included contributions of PCDFs and co-planar- and mono-*ortho*-PCBs, which were not independently associated with semen parameters. Although cross-sectional studies on PCBs have reported negative associations with semen parameters (Meeker and Hauser 2010), we did not find longitudinal associations between childhood serum concentrations of PCBs and semen parameters in our cohort.

Similar to our TCDD results, those of Mocarelli et al. (2008) showed that men from the Seveso cohort who were acutely exposed to very high levels of TCDD during childhood (ages 1–9 years) had impaired semen parameters. Specifically, they had a 27% decrease in sperm concentration (*p* = 0.025), a 20% decrease in sperm motility (*p* = 0.001), and a 39% decrease in total motile sperm count (*p* = 0.01) 22 years later, compared with men in the control group without acute high exposure. In contrast, the Seveso boys exposed to high levels of TCDD during puberty (ages 10–17 years) had higher total sperm count and total motile sperm count than did men in the control group. These results suggest a differential effect of TCDD by age at exposure. The OC measurements in the Russian Children’s Study reflect cumulative exposure up to age 8–9 years, whereas the boys in the Seveso cohort were exposed at a specific time point before age 10 years (mean age at exposure, 6.2 years); therefore, we can speculate that the Russian boys and this subset of Seveso boys were exposed before pubertal onset or very early during pubertal development. Both the Seveso study and our results suggest that the peripubertal period may be particularly susceptible to the deleterious effects of TCDD on adult semen parameters. In the Mocarelli et al. (2008) study, the median serum TCDD concentrations among the exposed group of children was 210 pg TEQ/g lipid and the control group had serum TCDD < 15 pg TEQ/g lipid. In contrast, for boys in our study, the median serum TCDD was 2.9 pg TEQ/g lipid, about 70-fold lower than exposed Seveso boys. Therefore, our results showed that childhood serum TCDD TEQ levels much lower than those measured in the Seveso study had a negative association with adult semen parameters. In addition, we found negative associations between childhood serum PCDD TEQs with sperm concentration, count and motile count, indicating that childhood exposure to other dioxins may also negatively affect semen parameters in adult life.

The period of sexual differentiation and reproductive tract organization during fetal development is highly sensitive to endocrine disrupting exposures which can affect reproductive-tract development and subsequent pubertal timing (Sharpe 2006). However, childhood and adolescence may also be vulnerable to such exposures due to the developmental changes of pubertal maturation that occur at these ages (Bin-Abbas et al. 1999; Grumbach 2002). Previously, we reported that higher peripubertal serum dioxins were associated with delayed pubertal onset and sexual maturity in the Russian cohort (Burns et al. 2016; Korrick et al. 2011). The proliferation and differentiation of Sertoli cells, the support cells of the seminiferous tubules, are peripubertal androgen-dependent processes that are critical for spermatogenesis (Sharpe et al. 2003). Dioxins can inhibit testosterone biosynthesis (Svechnikov et al. 2010), and may have direct testicular actions as the AhR is widely expressed in the testes (Schultz et al. 2003). AhR-mediated disruption of androgen activity could affect proliferation of the Sertoli cells and their subsequent differentiation, and pubertal maturation of the seminiferous tubules (Sharpe et al. 2003; Woodruff et al. 2010). These mechanisms could contribute to the observed decrease in sperm count in adults who were exposed to TCDD and PCDD TEQs as young children (Mocarelli et al. 2008).

Our findings are in agreement with animal data showing TCDD inhibition of testicular development and function during critical periods of reproductive-tract development, including fetal, neonatal (Arima et al. 2009; Faqi et al. 1998), pubertal (el-Sabeawy et al. 1998), and adult stages (Oguz et al. 2013; Sönmez et al. 2011). Moreover, childhood exposures to dioxins, furans, and PCBs have been shown to adversely affect other key maturational processes, such as somatic growth and pubertal timing in our cohort (Burns et al. 2011, 2016; Korrick et al. 2011).

Our study has several potential limitations. First, we did not measure prenatal exposure to OCs, when sexual differentiation and reproductive tract organization occur. Nevertheless, childhood is also a vulnerable developmental period. Second, we excluded boys with severe chronic illnesses at study entry. If their diseases were caused by or at least partially attributable to pre- or perinatal exposure to dioxins, furans, and/or PCBs, the association of these exposures with semen parameters may be underestimated in our analyses. Third, in our study, the boys’ median serum total TEQ concentrations were three times higher than the geometric mean in the U.S. National Health and Nutrition Examination Survey for males 12–19 years of age (no data were available for children < 12 years of age) (Patterson et al. 2008), and three times higher (using 1998 WHO total TEQs) than levels among similarly aged German boys (Link et al. 2005). This makes it difficult to investigate the effects of very low exposures in our cohort. However, despite this, our concentrations of TCDD were much lower than those in the Seveso study, which was used by the U.S. EPA in their dioxin risk assessment document (U.S. EPA 2009).

The strengths of our study include its prospective design and long-term serial follow-up of participants which minimizes the risk of reverse causation, the consistency in analysis of semen samples by the same laboratory technician which prevents interobserver variation, the comprehensive adjustment for possible confounding variables collected using physical examination and questionnaire data, and the availability of two semen samples on almost all participants (93%).

## Conclusions

Our results showed an association of peripubertal serum concentrations of TCDD with poorer semen parameters. Our results, along with toxicological evidence, suggest that peripubertal exposure to TCDD and dioxins may adversely impact adult semen parameters. We found this association at much lower TCDD concentrations than in the Seveso study, suggesting that moderate concentrations may also impact semen parameters and providing evidence that would be useful for risk assessment. Semen parameters are a marker of fertility and future studies on the impact of TCDD and dioxins on male fertility are warranted.

## Supplemental Material

(176 KB) PDF
