# Supplementary material for: A Longitudinal Study of Peripubertal Serum Organochlorine Concentrations and Semen Parameters in Young Men: The Russian Children’s Study
Source: Environ Health Perspect. 2016 Oct 7;125(3):460–6. doi: 10.1289/EHP25 (PMC5332179; doi:10.1289/EHP25)
Supplement: (176 KB) PDF [file EHP25.s001.acco.pdf]

Environ Health Perspect
DOI: 10.1289/EHP25

Note to readers with disabilities: *EHP* strives to ensure that all journal content is accessible to all readers. However, some figures and Supplemental Material published in *EHP* articles may not conform to 508 standards due to the complexity of the information being presented. If you need assistance accessing journal content, please contact ehponline@niehs.nih.gov. Our staff will work with you to assess and meet your accessibility needs within 3 working days.

Supplemental Material

A Longitudinal Study of Peripubertal Serum Organochlorine Concentrations and Semen Parameters in Young Men: The Russian Children's Study

Lidia Mínguez-Alarcón, Oleg Sergeyev, Jane S. Burns, Paige L. Williams, Mary M. Lee, Susan A. Korrick, Luidmila Smigulina, Boris Revich, and Russ Hauser

Table of Contents

Table S1. Mean sperm parameters among 133 men (contributing 256 semen samples) in the Russian Children's Study, by of serum dioxin, furans and PCBs

Table S1. Mean sperm parameters[a] among 133 men (contributing 256 semen samples) in the Russian Children's Study, by of serum dioxin, furans and PCBs.

	Volume (mL)	Sperm Concentration (mill/mL)	Total Sperm Count (mill)	Motile Sperm (%)	Total Motile Sperm Count (mill)
**TEQs (pg TEQ/g lipid)**					
TCDD					
Q1 [0.35-1.70]	2.75 (2.25, 3.25)	56.4 (44.3, 71.8)	129 (94.8, 175)	61.7 (58.5, 64.9)	61.7 (58.5, 64.9)
Q2 [1.77-2.45]	2.99 (2.56, 3.42)	52.4 (43.1, 63.6)	140 (110, 177)	65.5 (63.6, 67.4)	65.5 (63.6, 67.4)
Q3 [3.00-3.40]	2.47 (2.05, 2.89)	37.5 (27.3, 51.3)*	78.6 (53.3, 116)*	59.6 (56.5, 62.8)	59.6 (56.5, 62.8)
Q4 [4.40-5.80]	3.05 (2.42, 3.67)	34.0 (24.5, 47.1)*	86.2 (59.1, 126)	59.7 (56.1, 63.3)	59.7 (56.1, 63.3)
p, trend	0.78	0.005	0.02	0.11	0.02
PCDD TEQ					
Q1 [0.95-5.62]	3.14 (2.66, 3.62)	64.1 (52.4, 78.3)	171 (134, 218)	63.4 (60.5, 66.2)	107 (80.8, 142.3)
Q2 [5.69-8.42]	2.71 (2.26, 3.17)	37.3 (27.7, 50.1)*	89.0 (62.7, 126)*	59.8 (56.6, 63.0)	52.2 (35.4, 76.9)*
Q3 [8.68-13.3]	2.3 (1.92, 2.68)	41.3 (31.3, 54.3)*	79.2 (53.4, 117)*	63.2 (60.3, 66.0)	49.5 (32.3, 76.0)*
Q4 [13.7-36.0]	3.12 (2.5, 3.73)	38.7 (28.5, 52.5)*	103 (76.8, 139)*	60.4 (57.0, 63.8)	61.2 (43.8, 85.5)*
p, trend	0.68	0.02	0.02	0.44	0.02
PCDF TEQ					
Q1 [0.55-3.20]	3.10 (2.67, 3.53)	49.2 (35.8, 67.5)	135 (97.6, 187)	63.8 (61.2, 66.3)	85.4 (59.7, 122)
Q2 [3.29-4.66]	2.29 (1.85, 2.73)	42.5 (31.9, 56.6)	80.8 (54.7, 119)	59.2 (55.7, 62.7)	46.8 (30.4, 71.9)
Q3 [4.76-6.87]	2.98 (2.47, 3.48)	40.5 (32.6, 50.3)	103 (77.2, 138)	61.2 (58.3, 64.1)	62.3 (45.0, 86.4)
Q4 [7.10-50.6]	2.88 (2.30, 3.46)	44.9 (33.5, 60.1)	108 (77.4, 152)	62.4 (59.1, 65.8)	66.7 (46.1, 96.4)
p, trend	0.99	0.64	0.57	0.76	0.57
Co-PCB TEQ					
Q1 [0.52-4.63]	2.8 (2.2, 3.4)	53.6 (42.3, 67.9)	124 (92.8, 166)	63.2 (60.3, 66.1)	77.8 (56.6, 107)
Q2 [4.66-6.87]	2.9 (2.47, 3.3)	37.7 (26.9, 53.0)	98 (65.6, 146)	61.0 (58.1, 64.0)	58.7 (37.6, 91.5)
Q3 [6.88-9.97]	2.7 (2.2, 3.2)	37.4 (28.5, 49.2)	84 (58.9, 121)	61.6 (58.6, 64.7)	51.2 (34.5, 76.0)
Q4 [10.1-67.2]	2.8 (2.3, 3.4)	50.6 (39.7, 64.4)	121 (91.2, 160)	60.9 (57.4, 64.5)	72.3 (52.6, 99.5)
p, trend	0.91	0.76	0.74	0.40	0.64

Total TEQ					
Q1 [4.88-16.8]	3.01 (2.50, 3.52)	50.0 (36.2, 69.3)	129 (91.2, 182)	61.8 (58.6, 65.1)	78.5 (53.1, 116)
Q2 [17.0-21.4]	2.60 (2.14, 3.05)	40.0 (30.0, 53.4)	89.9 (60.8, 133)	61.6 (58.9, 64.4)	54.7 (36.0, 83.1)
Q3 [21.7-32.5]	2.79 (2.22, 3.36)	40.5 (32.6, 50.4)	89.7 (64.0, 126)	61.0 (58.1, 64.0)	54.0 (37.3, 78.1)
Q4 [33.3-107]	2.76 (2.28, 3.25)	44.7 (33.1, 60.5)	109 (80.7, 147)	61.7 (58.0, 65.4)	65.8 (46.8, 92.5)
p, trend	0.63	0.64	0.49	0.89	0.51
**Concentrations (pg/g lipid)**					
PCDD					
Q1 [37.6-115]	2.90 (2.46, 3.33)	51.2 (38.2, 68.6)	132 (95.1, 183)	64.6 (62.0, 67.1)	84.5 (59.3, 121)
Q2 [118-157]	2.51 (1.95, 3.07)	41.0 (31.5, 53.4)	81.4 (55.1, 120)	58.5 (55.4, 62.0)	46.8 (30.6, 71.5)
Q3 [158-200]	3.24 (2.73, 3.74)	38.3 (28.4, 51.6)	109 (77.8, 153.3)	63.4 (60.7, 66.0)	68.4 (46.8, 100)
Q4 [201-1237]	2.62 (2.15, 3.09)	47.3 (36.5, 61.4)	105 (79.4, 140)	60.3 (56.6, 64.0)	62.2 (45.1, 85.8)
p, trend	0.93	0.63	0.59	0.26	0.49
PCDF					
Q1 [14.4-29.2]	2.81 (2.34, 3.28)	51.5 (36.9, 71.8)	125 (86.7, 181)	63.7 (61.1, 66.3)	79.0 (53.4, 117)
Q2 [29.4-43.6]	2.59 (2.16, 3.02)	41.6 (32.2, 53.6)	90.4 (63.0, 130)	60.4 (57.1, 63.7)	53.5 (35.5, 80.7)
Q3 [44.5-63.0]	3.36 (2.73, 3.99)	38.7 (30.0, 50.0)	110 (82.1, 145)	60.2 (57.1, 63.4)	64.7 (46.7, 89.8)
Q4 [63.3-405]	2.50 (2.10, 2.91)	46.1 (35.2, 60.2)	100 (71.4, 141)	62.4 (59.2, 65.7)	61.7 (42.7, 89.1)
p, trend	0.88	0.56	0.55	0.56	0.52
Co-PCB					
Q1 [62.5-126]	2.57 (2.09, 3.05)	59.4 (45.7, 77.2)	127 (90.7, 179)	62.53 (59.37, 65.7)	78.6 (53.7, 115)
Q2 [130-184]	2.61 (2.22, 3.01)	38.5 (27.8, 53.4)	89.0 (62.3, 127)	61.32 (58.52, 64.13)	53.9 (36.4, 79.8)
Q3 [187-274]	3.10 (2.55, 3.66)	37.9 (28.9, 49.7)	96.7 (66.7, 140)	61.5 (58.6, 64.3)	58.6 (39.3, 87.3)
Q4 [275-965]	2.97 (2.43, 3.50)	44.3 (35.1, 55.9)	113 (86.1, 149)	61.4 (57.7, 65.1)	68.3 (49.6, 94.0)
p, trend	0.14	0.12	0.70	0.68	0.67
**Concentration (ng/g lipid)**					
ΣPCBs					
Q1 [58.3-151]	2.99 (2.45, 3.53)	50.6 (37.4, 68.5)	126 (90.5, 176)	62.6 (59.6, 65.7)	78.0 (53.8, 113)
Q2 [152-236]	2.54 (2.12, 2.97)	46.9 (34.8, 63.1)	103 (69.0, 153)	62.2 (59.7, 64.8)	63.1 (41.1, 96.8)

Q3 [239-352]	2.69 (2.26, 3.11)	34.6 (27.0, 44.4)	81.3 (60.7, 109)	61.6 (58.2, 65.0)	49.2 (35.0, 69.0)
Q4 [356-1500]	2.95 (2.33, 3.56)	44.2 (33.5, 58.3)	108 (76.6, 152)	59.7 (56.1, 63.2)	63.0 (43.4, 91.5)
p, trend	0.99	0.28	0.35	0.20	0.29

[a] Predicted means (95% confidence intervals) for each quartile from the mixed effect model, accounting for repeated measures of semen quality for each man, but unadjusted for any other covariates.
